# Correlates of group sex participation among men who have sex with men in Chongqing, Southwestern China

**DOI:** 10.1186/s12889-021-10607-0

**Published:** 2021-03-22

**Authors:** Jin Chen, Hui Fan, Huailiang Chen, Feifei Yao

**Affiliations:** 1 Department of Medical Records and Statistics, the People’s Hospital of Tongliang District, Chongqing, China; 2 Department of Preventive Medicine, North Sichuan Medical College, Nanchong, China; 3 Department of Infectious Diseases Prevention and Healthcare, the People’s Hospital of Sichuan Tianfu New Area, Chengdu, China; 4 Department of STD/HIV Control and Prevention, Sichuan Tianfu New Area Center for Disease Control and Prevention, Chengdu, China

**Keywords:** Group sex participation, Correlates, MSM, CIE

## Abstract

**Background:**

Findings from specific countries indicated group sex was common among men who have sex with men (MSM), and men who reported group sex participation were at increased risk of human immunodeficiency virus (HIV)/sexually transmitted infections (STIs). The purpose of the current analysis was to describe the prevalence and correlates of group sex participation among a community-based sample of MSM in Chongqing, southwestern China.

**Methods:**

Convenience sampling method was used to recruit participants and data were collected through an anonymous questionnaire. Logistic regression analysis was employed to identify correlates of group sex participation.

**Results:**

Overall, 1151 eligible participants were enrolled in the study. 14.7% of MSM reported participating in group sex in their lifetime, and 5.8% reported group sex participation in the prior 6 months. Factors positively associated with group sex participation in both the prior 6 months and the lifetime included: monthly income ≥3000 Yuan (adjusted odds ratios [aOR] = 3.67, 95% confidence interval [CI] 1.25–10.75; aOR = 2.30, 95% CI 1.21–4.35), initiating anal sex before 18 years old (aOR = 1.86, 95% CI 1.03–3.34; aOR = 2.00, 95% CI 1.31–3.05), using gay apps to seek sex partners (aOR = 7.41, 95% CI 2.57–21.33; aOR = 9.75, 95% CI 4.92–19.33), recreational drug use (aOR = 10.10, 95% CI 5.52–18.49; aOR = 4.75, 95% CI 3.20–7.05) and having condomless internal ejaculation (CIE) (aOR = 3.66, 95% CI 2.01–6.68; aOR = 1.61, 95% CI 1.11–2.35). Factors only associated with group sex participation in the lifetime were older age (age between 26 and 35 years old: aOR = 2.06, 95% CI 1.30–3.26; age > 35 years old: aOR = 1.95, 95% CI 1.10–3.46) and history of STIs (aOR = 2.51, 95% CI 1.37–4.62).

**Conclusions:**

The results of this study suggested that group sex participation was a potentially risky context for acquisition and transmission of HIV/STIs. Close attention should be given to MSM who participated in group sex, and appropriate risk reduction interventions should be developed specific to this subgroup of MSM.

**Supplementary Information:**

The online version contains supplementary material available at 10.1186/s12889-021-10607-0.

## Background

A growing body of evidence has indicated that men who have sex with men (MSM) are one of the groups seriously affected by human immunodeficiency virus (HIV) in China. National estimates on the HIV epidemic showed that there were 780,000 people living with HIV (PLHIV) in China by the end of 2011, 17.4% of which were attributed to homosexual transmission, while sexual contact between men only accounted for 14.7 and 11.0% of PLHIV in 2009 and 2007, respectively [1, 2]. Additionally, recent update on the HIV epidemic in China revealed homosexual contacts accounted for 25.5% of new HIV diagnoses in 2017 [3], which was doubled since 2007 (12.2%) [2].

Findings from Australia, United Kingdom and United States have documented that group sex was a common behavior among MSM [4–7], and men were more likely to report recreational drug use and unprotected anal intercourse (UAI) with casual, HIV unknown or HIV serodiscordant partners in the setting of group sex [7–9]. Thus, men participating in group sex were found at higher risk of infection with and transmission of HIV/sexually transmitted infections (STIs) [9–11]. A small number of reports from coastal metropolises of China have addressed the issue of group sex among MSM [12–14]. However, studies examining factors associated with group sex participation are rather limited in China. Of the existing literature among MSM in China, an online survey from 2014 found men living with HIV, practicing condomless anal intercourse and using mobile phone application were more likely to engage in group sex in 12 the prior months [15]. Another cross-sectional study mainly discussed the correlation between poppers use and lifetime group sex engagement among MSM in the neighboring Sichuan province, southwestern China [16].

Chongqing is the largest provincial municipality located in southwest China. It has a total population of 34.04 million and an area of 82,400 km^2^ [17]. In the past decades, Chongqing municipality has obtained brilliant achievements in social culture, commerce, economic development and city infrastructure construction [17], which made the city a comprehensive traffic hub and the economic and political center of southwest China. Therefore, a great number of migrant and floating populations nationally were attracted to live and work in this city. Chongqing reportedly has a large population of young MSM. They are sexually active and frequently engaging in sexual risk behaviors [18, 19]. Meanwhile, regular HIV testing level among the subpopulation was low [19, 20]. Thus, the highest HIV prevalence was observed among MSM in Chongqing [21].

In consideration of the severity of HIV epidemic among MSM in Chongqing and the fact that group sex participation within the subpopulation could potentially fuel the severe epidemic, we sought to describe factors correlated with group sex participation, as well as its relationship with risky behaviors, among a community-based sample of MSM in this under-researched area. Understanding the factors underlying group sex participation among MSM might inform future interventions for this subpopulation.

## Methods

### Study design and participants

The data used in the current analysis were derived from a larger cross-sectional study aiming to describe recreational drug use, sexual risk behaviors and STIs among MSM in Chongqing. Convenience sampling method was employed to recruit MSM participants through the largest community-based organization (CBO) in Chongqing municipality from March 2019 to February 2020. MSM were requested to join the study when they attended the CBO for voluntary counseling and testing (VCT) services. Firstly, potential participants were informed of the purpose, procedures, contents and confidentiality issues of the study, and MSM were guaranteed that they could get free VCT services, no matter they participated in the study or not. CBO staff subsequently checked the eligibility of MSM and asked qualified participants to provide a written informed consent. And then information of participants was collected through a self-administered questionnaire after all these procedures were completed. To obtain necessary sample size, enrolled MSM were encouraged to refer their peers.

Men were eligible for the study if they were at least 18 years old, had sex with males in the prior 12 months and were willing to provide written informed consent. Enrolled participants were paid 25 Yuan (~ 4 American dollar) each as transportation fees. This study was carried out with approval of the ethics committee of the people’s hospital of Chongqing Tongliang District and the people’s hospital of Sichuan Tianfu New Area.

### Data collection

A self-administered, structured questionnaire was developed to collect information from participants anonymously, and the selected variables for the present analysis could be viewed in Supplementary file 1. In brief, MSM participants were asked to provide their socio-demographics and information on recreational drug use behavior, sexual orientation, main sexual role with males, sexual behaviors, HIV testing and history of STIs.

The main outcome of this study was group sex participation, which was assessed by the questions “Have you ever had sex with three or more men during a single sexual encounter?” and “Have you had sex with three or more men during a single sexual encounter in the prior 6 months?”

### Data analysis

Frequencies and proportions were used to present categorical variables, and median and interquartile range (IQR) were used to describe continuous variables. Factors correlated with group sex participation were evaluated using logistic regression analysis. Univariate logistic regression analysis was first conducted to examine the odds of factors correlated with group sex participation. Factors significant at *P* < 0.05 were subsequently included into the multivariate stepwise logistic regression model to obtain adjusted odds ratios (aORs) and 95%CI. Significance level was set at *P* < 0.05. All analysis was conducted by SPSS 16.0 (SPSS, Inc., Chicago, IL, USA).

## Results

### Socio-demographics, drug use and sexual behaviors of participants

Overall, 1151 MSM were included in the present analysis. The demographic characteristics of participants are presented in Table 1. The median age of enrolled MSM was 28 (IQR 24–34) years old. The majority of participants were unmarried (79.5%), Chongqing residents (74.4%) and solo business owners or service providers (66.9%). Most MSM had at least a college education level (68.9%) and a monthly income of ≥3000 Yuan (81.1%).

**Table 1:** Sociodemographic characteristics of participants (*n* = 1151)

Variables	n	%
**Age (years)**		
≤ 25	410	35.6
26–35	491	42.7
> 35	250	21.7
Median (IQR)	28 (24–34)	
**Marital status**		
Never married	915	79.5
Married	162	14.1
Divorced or widowed	74	6.4
**Residence in Chongqing**		
Yes	856	74.4
No	295	25.6
**Education**		
Senior high school and below	358	31.1
College and above	793	68.9
**Occupation**		
Enterprise, public institution or government	186	16.2
Solo business owners or service providers	770	66.9
Retired, unemployed or students	195	16.9
**Monthly income (Yuan)**		
< 3000	217	18.9
≥ 3000	934	81.1

Of the participants enrolled, 18.9% (218) reported recreational drug use in the prior 6 months. The most frequently reported drugs were rush poppers (17.7%), methamphetamine (4.5%) and magu (1.0%). Other drugs used among MSM in Chongqing were less than 1.0% (Table 2).

**Table 2:** Behaviors and health outcomes among MSM (*n* = 1151)

Variables	n	%
**Drug used in the prior 6 months**		
Any drug use	218	18.9
Rush poppers	204	17.7
Methamphetamine	52	4.5
Magu	12	1
Capsule zero	3	0.3
Ketamine	3	0.3
Other drugs	5	0.4
**Sexual orientation**		
Homosexual	768	66.7
Bisexual	320	27.8
Uncertain	63	5.5
**Main sexual role with males**		
Insertive anal intercourse	383	33.3
Receptive anal intercourse	265	23.0
Versatile anal intercourse	503	43.7
**Initiating anal sex before 18 years old**		
Yes	241	20.9
No	910	79.1
**Using gay apps to seek sex partners**		
Yes	774	67.2
No	377	32.8
**Number of male sex partners in the prior 6 months**		
< 2	462	40.1
≥ 2	689	59.9
**Condomless internal ejaculation during anal intercourse in the prior 6 months**		
Yes	480	41.7
No	671	58.3
**Group sex**		
Group sex in the prior month	31	2.7
Group sex in the prior 6 months	67	5.8
Group sex in the lifetime	169	14.7
Group sex after drug use	85	7.4
**HIV testing in the prior 6 months**		
Yes	428	37.2
No	723	62.8
**History of STIs**		
Yes	72	6.3
No	1079	93.7

Table 2 also demonstrates information on sexual behaviors, HIV testing and STIs of MSM participants. Among all participants included, the majority of (66.7%) MSM self-identified as homosexuality. One-fifths (20.9%) reported initiating their anal sex before 18 years old. Over two-thirds (67.2%) reported using cell phone-based applications especially for MSM (gay apps) to seek male sex partners. Almost three-fifths (59.9%) had more than one sex partner in the prior 6 months, and 41.7% of MSM reported having condomless internal ejaculation (CIE) during anal intercourse in the prior 6 months. Respectively, 2.7, 5.8 and 14.7% of MSM reported having participated in group sex in the prior month, in the prior 6 months and in their lifetime. In this sample, less than two-fifths (37.2%) reported having tested HIV recently and 6.3% reported a history of STIs.

### Factors correlated with group sex participation in the prior 6 months

In the univariate analysis, variables significantly correlated with group sex participation in the prior 6 months (*P* < 0.05) were: monthly income ≥3000 Yuan, initiating anal intercourse before 18 years old, using gay apps to seek sex partners, recreational drug use, practicing CIE during anal intercourse and having a history of STIs (Table 3).

**Table 3:** Univariate analysis of factors correlated with participation in group sex (*n* = 1151)

Variables	Participation in group sex in the prior 6 months		Lifetime participation in group sex	
	OR(95% CI)	***P*** value	OR(95% CI)	***P*** value
**Age (years)**				
≤ 25	1		1	
26–35	1.38 (0.79–2.42)	0.262	2.05 (1.39–3.01)	< 0.001
> 35	0.93 (0.45–1.93)	0.854	1.21 (0.74–1.98)	0.451
**Residence**				
Chongqing	1		1	
Other	0.93 (0.54–1.63)	0.811	1.01 (0.70–1.47)	0.950
**Marital status**				
Single	1		1	
Married or cohabitating	1.19 (0.63–2.27)	0.588	1.35 (0.88–2.09)	0.171
**Monthly income (Yuan)**				
< 3000	1			
≥ 3000	3.85 (1.39–10.70)	0.010	2.89 (1.63–5.09)	< 0.001
**Initiating anal sex before 18 years old**				
No	1		1	
Yes	2.63 (1.14–6.08)	0.023	2.21 (1.55–3.15)	< 0.001
**Using gay apps to seek sex partners**				
No	1		1	
Yes	8.26 (2.98–22.88)	< 0.001	9.49 (4.94–18.21)	< 0.001
**Recreational drug use in the prior 6 months**				
No	1		1	
Yes	16.04 (9.03–28.48)	< 0.001	6.46 (4.54–9.19)	< 0.001
**Condomless internal ejaculation during anal intercourse in the prior 6 months**				
No	1		1	
Yes	4.12 (2.37–7.18)	< 0.001	1.64 (1.18–2.27)	0.003
**HIV testing in the prior 6 months**				
No	1		1	
Yes	1.40 (0.85–2.30)	0.187	0.89 (0.63–1.25)	0.508
**History of STIs**				
No	1		1	
Yes	2.52 (1.19–5.31)	0.016	3.70 (2.22–6.17)	< 0.001

*CI* confidence interval, *OR* odds ratio

In the multivariate analysis, monthly income ≥3000 Yuan (aOR = 3.67, 95% CI 1.25–10.75), initiating anal intercourse before 18 years old (aOR = 1.86, 95% CI 1.03–3.34), using gay apps to seek sex partners (aOR = 7.41, 95% CI 2.57–21.33), recreational drug use (aOR = 10.10, 95% CI 5.52–18.49) and practicing CIE during anal intercourse (aOR = 3.66, 95% CI 2.01–6.68) were significantly associated with group sex participation in the prior 6 months (*P* < 0.05) (Table 4).

**Table 4:** Multivariate analysis of factors correlated with participation in group sex (*n* = 1151)

Variables	Participation in group sex in the prior 6 months		Lifetime participation in group sex	
	aOR(95%CI)	***P*** value	aOR(95%CI)	***P*** value
**Age (years)**				
≤ 25			1	
26–35			2.06 (1.30–3.26)	0.001
> 35			1.95 (1.10–3.46)	0.017
**Monthly income (Yuan)**				
< 3000	1		1	
≥ 3000	3.67 (1.25–10.75)	0.018	2.30 (1.21–4.35)	0.011
**Initiating anal sex before 18 years old**				
No	1		1	
Yes	1.86 (1.03–3.34)	0.039	2.00 (1.31–3.05)	0.001
**Using gay apps to seek sex partners**				
No	1		1	
Yes	7.41 (2.57–21.33)	< 0.001	9.75 (4.92–19.33)	< 0.001
**Recreational drug use**				
No	1		1	
Yes	10.10 (5.52–18.49)	< 0.001	4.75 (3.20–7.05)	< 0.001
**Condomless internal ejaculation during anal intercourse in the prior 6 months**				
No	1		1	
Yes	3.66 (2.01–6.68)	< 0.001	1.61 (1.11–2.35)	0.013
**History of STIs**				
No	1		1	
Yes	1.16 (0.49–2.77)	0.735	2.51 (1.37–4.62)	0.003

*CI* confidence interval, *aOR* adjusted odds ratio

### Factors associated with group sex participation in the lifetime

Table 3 also shows univariate analysis of factors significantly associated with group sex participation in the lifetime (*P* < 0.05), including: older age, monthly income ≥3000 Yuan, initiating anal intercourse before 18 years old, using gay apps to seek sex partners, recreational drug use, practicing CIE during anal intercourse and having a history of STIs.

Factors associated with increased odds of lifetime group sex participation in the multivariate logistic regression model were: older age (age between 26 and 35 years old: aOR = 2.06, 95% CI 1.30–3.26; age > 35 years old: aOR = 1.95, 95% CI 1.10–3.46), monthly income ≥3000 Yuan (aOR = 2.30, 95% CI 1.21–4.35), initiating anal intercourse before 18 years old (aOR = 2.00, 95% CI 1.31–3.05), using gay apps to seek sex partners (aOR = 9.75, 95% CI 4.92–19.33), recreational drug use (aOR = 4.75, 95% CI 3.20–7.05), practicing CIE during anal intercourse (aOR = 1.61, 95% CI 1.11–2.35) and having a history of STIs (aOR = 2.51, 95% CI 1.37–4.62) (Table 4).

## Discussion

The current study aimed to describe the prevalence and correlates of group sex participation among a community-based sample of MSM in Chongqing municipality, where the highest HIV prevalence was observed among MSM in the country. Of the 1151 participants included in this study, 14.7 and 5.8% reported having participated in group sex in the lifetime and in the prior 6 months, respectively. The prevalence was comparable with results from online survey and metropolises elsewhere in China [15, 22, 23], but significantly lower than reports in countries, like United States and Australia [6, 7, 24]. The disparity may partly reflect differences in culture and attitudes towards non-mainstream sexual activities.

In the multivariate model, demographic characteristic variables, such as age and monthly income, were found to be positively correlated with group sex participation, which was consistent with prior findings [4, 16]. Older MSM might be more active in sexual sensation seeking and adventurous in sexual behaviors than their younger counterparts [25]. Meanwhile, prior research found group sex was associated with substances use [24], and substances use might be quite an expenditure to lower-income MSM. Hence, it is understandable that men with older age and higher income were more likely to participate in group sex. Additionally, our study found that earlier anal intercourse initiation was significantly associated with group sex participation. Similar to findings in the current study, prior studies indicated that MSM who initiated their sex experience earlier were more likely to practice risky behaviors in their later life [26, 27]. Therefore, MSM with such characteristics should be a priority for further interventions towards group sex participation.

Corroborating the prior findings [15, 16], we found seeking sex partners through gay applications was positively associated with participating in group sex. In recent years, the use of gay applications, such as Blued and Aloha, has been increasingly popular among MSM in China [28–30]. These applications use the global positioning system (GPS) to identify nearby peers, which have rapidly expanded social networking and partner-seeking opportunities among MSM. Thus, men could effectively arrange or participate in group sex by using these gay applications. Moreover, MSM who reported recreational drug use were more likely to participate in group sex. It has been previously documented that recreational drug use, especially rush poppers and methamphetamine use, was prevalent among MSM who engaged in group sex [7, 24]. Recreational drugs were reported to increase libido, maintain erection, prolong sexual activity and maximize sexual pleasure [31, 32]. This may explain the reason for higher prevalence of group sex participation among recreational drug users observed in this study.

In accordance with findings from previous studies, we found participating in group sex was associated with increased odds of practicing CIE during anal intercourse. Although it is not possible to determine from this study whether the risk behavior occurred in the context of group sex, existing literature indicated that MSM who attended sex parties practiced higher levels of UAI [7, 8]. Given the severity of HIV/STIs among MSM in Chongqing, the risky combination of group sex participation and CIE during anal intercourse could potentially fuel the epidemics among this subpopulation. Moreover, we found MSM ever participating in group sex were more likely to report history of STIs. The relationship between group sex participation and STIs was discussed in previous studies [33–35]. A high partner turnover rate and low condom use rate in the context of group sex might contribute to infection with and transmission of STIs among men participating in group sex. Therefore, our findings highlight the need for interventions on group sex participation and associated risky behaviors.

This study has a few limitations. Firstly, our study may be vulnerable to selection bias, as all participants in this study were recruited by a CBO. Secondly, data on recreational drug use, sexual behaviors and history of STIs were still sensitive in China, and the results might be subjected to social desirability bias. Thirdly, our findings were based on a self-administered questionnaire and recall bias might exist. Lastly, due to the nature of cross-sectional study, we can not infer causal relationships between group sex participation and associated factors. Future research in China should be conducted to collect an event-level behavioral data related to group sex, which may contribute to better understanding of the risks posed by group sex participation.

## Conclusions

Although the prevalence of group sex participation among MSM in Chongqing was not comparable with results from western countries, still we found MSM participating in group sex were more likely to report recreational drug use and CIE during anal intercourse. Thus, group sex might be a risky setting, posing an elevated risk for HIV infection and other STIs. More attention should be paid to group sex among MSM, and appropriate risk reduction interventions should be developed specific to this subgroup of MSM.

## Supplementary Information

**Additional file 1.** Questionnaire for MSM in Chongqing. Questionnaire for MSM in Chongqing.

## Data Availability

The datasets used during the current study are available from the corresponding author on reasonable request.

## Abbreviations

MSM: Men who have sex with men
HIV: Human immunodeficiency virus
STIs: Sexually transmitted infections
aOR: adjusted Odds ratio
OR: Odds ratio
CI: Confidence interval
CIE: Condomless internal ejaculation
PLHIV: People living with HIV
UAI: Unprotected anal intercourse
CBOs: Community-based organizations
VCT: Voluntary counseling and testing
IQR: Interquartile range
CDCs: Centers for disease control and prevention
GPS: Global positioning system
