# Supplementary material for: Correlates of group sex participation among men who have sex with men in Chongqing, Southwestern China
Source: BMC Public Health. 2021 Mar 22;21:561. doi: 10.1186/s12889-021-10607-0 (PMC7983368; doi:10.1186/s12889-021-10607-0)
Supplement: Supplementary file 1 — Additional file 1. Questionnaire for MSM in Chongqing. Questionnaire for MSM in Chongqing. [file 12889_2021_10607_MOESM1_ESM.doc]

**Questionnaire for MSM in Chongqing**

| **Part 1 Socio-demographic characteristics.** |
| --- |
| - 1. Your age: ________(years) |
| 1-2 Marital status:  (1) Never married (2) Married with woman (3) Divorced or Widowed (4)Others: ________ |
| 1-3 Province of permanent residence (Hukou):  (1) Chongqing municipality (2) Other provinces (_________) |
| 1-4 The highest education attained:  (1) Primary school or lower (2) Junior high school (3) Senior high school (or equal academic qualification) (4) College or above |
| 1-5 Your occupation:  (1) Enterprise, public institution or government (2) Service industry, solo business owner (3) Retired, unemployed or student  1-6 Your individual monthly income:  (1) <3000 (2) 3000～5999 (3) 6000～9999 (4) ≥10000 |

| **Part 2：Drug use, sexual orientation and sexual role with males**  2-1 Which of the following drug(s) have you used in the prior 6 months?  (1) Rush poppers (2) Capsule zero (3) Methamphetamine (4) Ecstasy (5) Magu  (6) Ketamine (7) Happy water (8) GHB (9) Cannabis (10) Bath salt (11) Red crystal meth (12) Heroin (13) Other drugs (_________)  2-2 What is your sexual orientation?  (1) Homosexual (2) Bisexual (3) Heterosexual (4)Uncertain  2-3 What is your main sexual role with males?  (1) Insertive (2) Receptive (3) Versatile  **Part 3：Sex behaviors and health outcomes.**  3-1 Your age at first anal intercourse : ________ (years)  3-2 What is your main route to seek male sex partners?  (1) Social networking software (2) Bars/ dance halls (3) Parks (4) Public bathrooms (5) Hotels (6) Others (_________) |
| --- |
| 3-3 Have you used the following phone applications to seek sex partners in the prior 6 months?  (1) Blued (2) Jack’d (3) Grindr (4) Scruff (5) Aloha (6) Zank (7) Wechat (8) QQ (9) Others (_________)  3-4 The number of male sex partners you have in the prior 6 months: (_________)  3-5 Have you practiced condomless internal ejaculation during anal sex in the prior 6 months?  (1) Yes (1) No  3-6 Have you participated in group sex (had sex with three or more men during a single sexual encounter) in the prior month?  (1) Yes (1) No  3-7 Have you participated in group sex in the prior 6 months?  (1) Yes (1) No  3-8 Have you ever participated in group sex in your lifetime?  (1) Yes (1) No  3-9 Have you ever participated in group sex after drug use?  (1) Yes (1) No  3-10 Do you tested HIV in the prior 6 months?  (1) Yes (1) No  3-11 Have you ever diagnosed any sexually transmitted infections?  (1) Yes (1) No |

(End of the survey, thanks for your participation!)
